# Novel lanthanide-labeled metal oxide nanoparticles improve the measurement of in vivo clearance and translocation

**DOI:** 10.1186/1743-8977-10-1

**Published:** 2013-01-10

**Authors:** Aamir D Abid, Donald S Anderson, Gautom K Das, Laura S Van Winkle, Ian M Kennedy

**Affiliations:** 1 Department of Mechanical and Aerospace Engineering, University of California, Davis, CA 95616, USA; 2 Center for Health and the Environment, University of California, Davis, CA, 95616, USA

**Keywords:** Nanoparticles, Translocation, Metal oxide, Lanthanide, ICP-MS

## Abstract

The deposition, clearance and translocation of europium-doped gadolinium oxide nanoparticles in a mouse lung were investigated experimentally. Nanoparticles were synthesized by spray flame pyrolysis. The particle size, crystallinity and surface properties were characterized. Following instillation, the concentrations of particles in organs were determined with inductively coupled plasma mass spectrometry. The protein corona coating the nanoparticles was found to be similar to the coating on more environmentally relevant nanoparticles such as iron oxide. Measurements of the solubility of the nanoparticles in surrogates of biological fluids indicated very little propensity for dissolution, and the elemental ratio of particle constituents did not change, adding further support to the contention that intact nanoparticles were measured. The particles were intratracheally instilled into the mouse lung. After 24 hours, the target organs were harvested, acid digested and the nanoparticle mass in each organ was measured by inductively coupled plasma mass spectrometry (ICP-MS). The nanoparticles were detected in all the studied organs at low ppb levels; 59% of the particles remained in the lung. A significant amount of particles was also detected in the feces, suggesting fast clearance mechanisms. The nanoparticle system used in this work is highly suitable for quantitatively determining deposition, transport and clearance of nanoparticles from the lung, providing a quantified measure of delivered dose.

## Introduction

Exposure to particles in the fine and ultrafine size range contribute to the development and exacerbation of respiratory diseases and cardiovascular events in humans [1]. This is especially true for susceptible populations such as children with asthma [2,3]. In 1998, the National Academy of Sciences issued a report on Research Priorities for Airborne Particulate Matter [4] that defined deposition patterns and fate of particles in the respiratory tract, especially for individuals who are susceptible to particulate matter (PM), as a high priority research area. This includes the study of deposition in adults with models of respiratory disease (such as asthma and COPD) – and also in the developing lung. The current study outlines a methodologic approach to address these issues in future studies. This approach involves actual measurement of deposited and retained dose. This is an important advance because, while the hypothesized effect of a remodeled asthmatic airway or the smaller airways of a young child can be used to theoretically predict increased deposition of fine and ultrafine PM, and may also impact clearance, to date there has not been a viable, highly efficient, low cost alternative that will allow this to be directly measured in an animal model. We propose the use of a new model particle to address this issue.

Several mechanisms have been proposed to describe toxicological responses due to nanoparticle exposure including local inflammation of the lung that further develops into systemic inflammation, and translocation of particles to organs such as the heart or brain where toxic effects are manifested [5]. The deposition, translocation and bio-distribution of nanoparticles, either intravenously injected or instilled intratracheally, have been reviewed previously [6,7].

Previous studies of translocation have made use of engineered nanoparticles with properties that permit them to be tracked in animal tissues, or to be imaged. Radioactive tags have been commonly used for this purpose, including technetium-99 m [8]. The tags are chemically attached to nanoparticles but may become separated from the nanoparticles and give a false indication of location. Alternative radio-labeled materials have been used – Kreyling [9], for example, used radioactive iridium nanoparticles to study translocation in an animal. In an alternative approach, Choi et al. [10] used quantum dots and fluorescently labeled polystyrene particles to study early particle translocation in the first hour after instillation. The study was confined to the lung and lymphatic system where concentrations were quite high and fluorescence imaging was feasible for early time-points following exposure. This approach is not likely to be successful in detecting translocation to more peripheral organs where concentrations, and hence fluorescence signals, are likely to be close to the limit of detection and confounded by background auto-fluorescence from tissues.

In both these examples, in common with other reported studies of particle clearance and translocation, the particles do not model well materials that are environmentally or technologically relevant; the coating of proteins on the surfaces of particles that are presented to cells are unlikely to represent more relevant materials such as metal oxides. Humans are commonly exposed to metal oxide nanomaterials. For example metal oxide nanomaterials can be associated with fugitive dust. One documented source of fugitive dust is the erosion of mine tailings in high winds and arid conditions. Metal oxide nanomaterials generated from these locations can travel great distances, affecting populations downwind of the site [11]. Ultrafine metal oxides are emitted from combustion sources. Kennedy [12] noted that metal oxides arise from components of engine fuels and lubricants, as well as engine components. The oxides of iron and zinc are the most common metals emitted from engines, often in association with other aerosol materials. Finally, metal oxide nanoparticles are finding increasing application in nanotechnologies that include environmental sensing [13]. Inorganic lanthanide-based nanoparticles are used in a range of biotechnologies [14]. Aerosol synthesis methods [15] offer an attractive and economical route to the production of these materials in large amounts, leading to a growing concern with regard to occupational exposures via inhalation during production and handling.

We propose an alternative to the extant approaches for evaluating translocation and clearance *in vivo*. In the current study, oxides of gadolinium were synthesized in an aerosol flame reactor. A key advantage in using metal oxide particles for translocation studies compared to radiolabeled particles, inert Au or quantum dots etc., is that the protein corona adsorbed onto the particle will be similar to the corona on naturally occurring non-carbonaceous PM. Doping with rare earth elements provides a convenient method of labeling, and also provides very high sensitivity for the detection of the nanoparticles using inductively-coupled plasma mass spectrometry (ICP-MS). Gadolinium oxide is an ideal host material to support strong optical emission from the lanthanide Europium ion, and has a similar zeta potential to other commonly occurring or engineered metal oxide nanomaterials. Hence, in addition to the relevance to some of the technological applications of Gadolinium oxides *per se*, Eu:Gd_2_O_3_ is a good surrogate material for tracking a metal oxide nanoparticle delivered to an animal.

The aerosol synthesis route is ideal for providing sufficient aerosol for a future inhalation study. An aerosol reactor provides sufficient process control to assure repeatable composition and size of the particles at concentrations that are environmentally relevant and that can be delivered for extended periods. Particle size can be refined using impactors to reduce the spread of droplet sizes, and mobility classifiers can reduce the size spread of the final metal oxide particles [16]. With suitable conditions, confounding gas-phase compounds such as NO_x_ can be reduced to insignificant levels.

The goals of this study were two-fold: 1) to provide a proof-of-concept of the usefulness of nanoparticles of this type for studying lung clearance rates and translocation over longer time-scales (on the order of a day); and 2) to define the methodology for use of these particles quantitatively *in vivo*.

## Results

### Particle characterization

**Figure 1:**
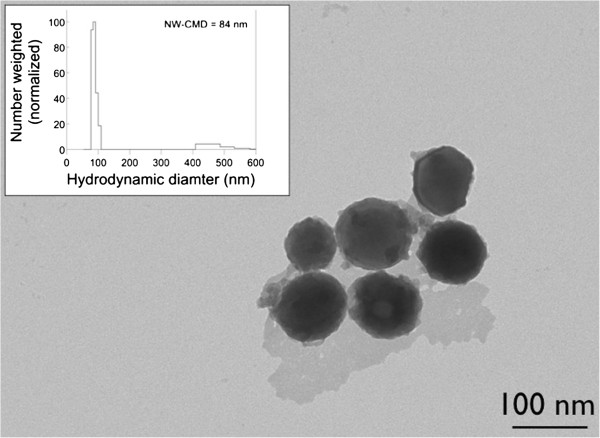
**Transmission electron microscopy image of flame synthesized Eu:Gd**_2_**O**_3 _**nanoparticles.** Inset: number weighted (NW) particle size distributions determined by dynamic light scattering. CMD, count median diameter.

A representative TEM image of particles synthesized by flame spray pyrolysis is shown in Figure 1. The particles are approximately spherical with a majority of the particles of approximately 80–100 nm diameter; a few larger clusters (~500 nm) were also observed on the grid. Particle sizes can be fine-tuned into specific size ranges by altering spray droplets sizes and the concentrations of precursors in solution. Further refinement can be achieved using impactors to minimize the size distribution of the precursor droplets, and mobility classification to eliminate aggregates [16]. Aggregation seen in the image is likely due to a sampling artifact that arises from the drying of the nanoparticle solution on the TEM grid. The dynamic light scattering (DLS) number-weighted size distribution is shown in the inset; the count mean hydrodynamic diameter is 84 nm, in good agreement with that observed by TEM.

**Figure 2:**
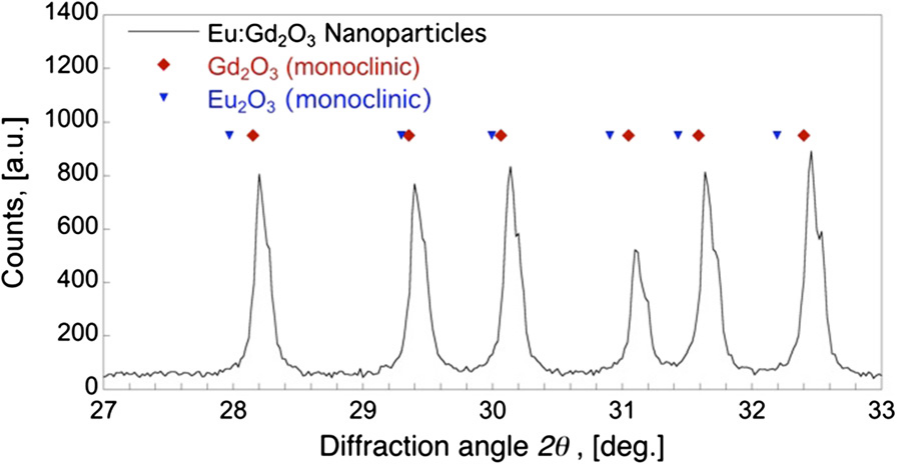
**Powder x-ray diffraction spectra for Eu:Gd**_2_**O**_3 _**flame synthesized nanoparticles.** Diamonds correspond to major peaks for monoclinic gadolinium oxide (PDF 00-042-1465) and inverted triangles correspond to monoclinic europium oxide (PDF 00-034-0072).

The XRD spectrum for the particles in Figure 2 shows peaks corresponding to monoclinic Gd_2_O_3_ ; the absence of peaks at 32.2° and 31.4° (major peaks for monoclinic Eu_2_O_3_) suggests that the Gd and Eu atoms are well-mixed within the particle. The Eu ion substitutes for the Gd ion, forming a well-ordered crystal.; separate phases of Gd or Eu are not evident in the XRD results. The BET measurement indicated that the nanoparticles have a specific surface area of 11.5 ± 1.0 m^2^ g^−1^. The BET effective diameter is determined by,

(1) $$d_{\mathrm{BET}}=\frac{6}{A_{S}\rho }$$

where *A*_*s*_ is the specific surface area and is the particle density equivalent to that of bulk gadolinia of 7.41 g cm^-3^[17], leading to a *d*_*BET*_ of 70.4 nm. The crystalline diameter *d*_*XRD*_, calculated using Scherrer’s formula (described in the Method section), was 66.5 nm and agrees well with the BET specific diameter.

#### Particle dissolution

**Figure 3:**
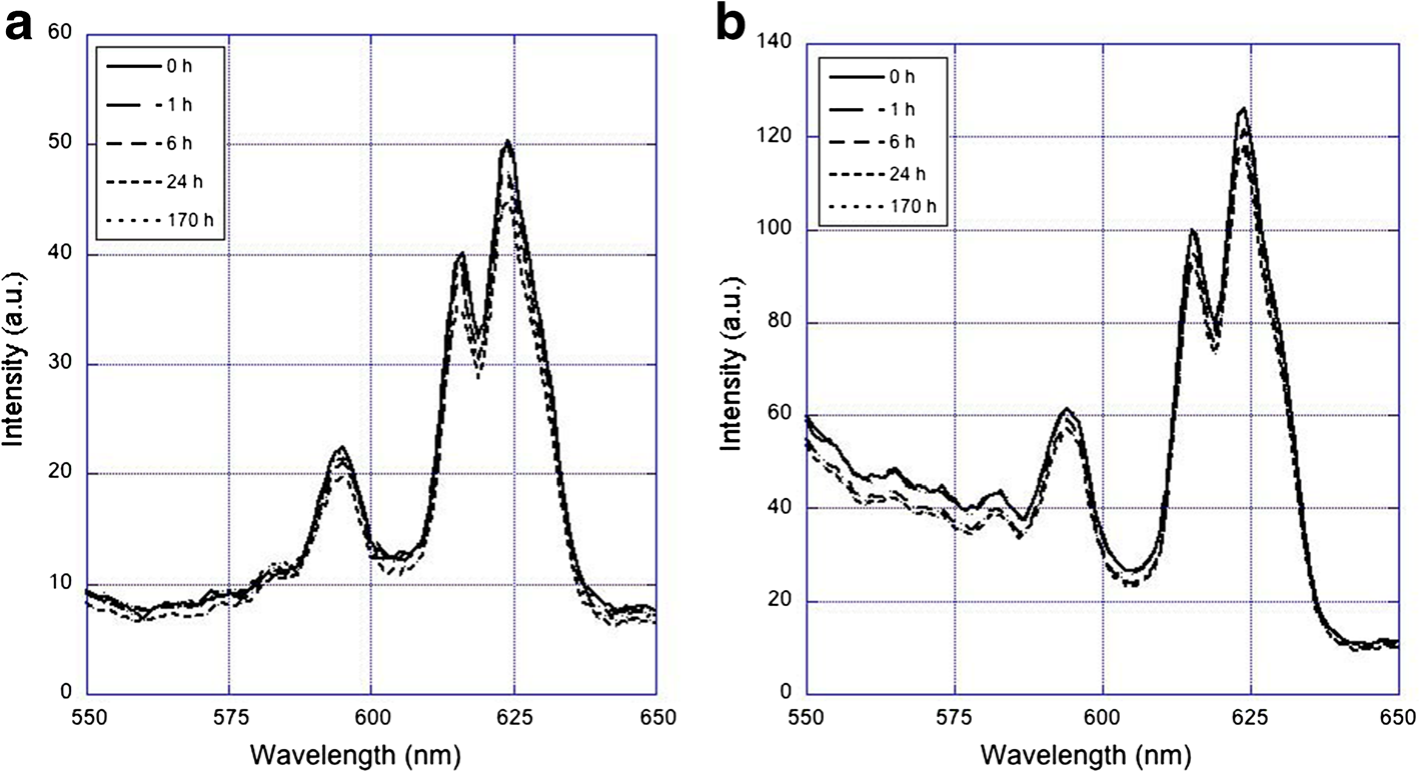
**Photoluminescence spectrum of emission of Eu: Gd**_2_**O**_3 _**suspended in (a) lung serum, (b) PBS at pH 5 for periods up to 170 hours.** The emission peaks at 590, 613, and 623 correspond to ^*5*^*D*_*0*_ → *F*_*2*_*,*^*5*^*D*_*0*_ → ^*7*^ *F*_*1*_ and ^*5*^*D*_*0*_ → ^*7*^ *F*_3_ transitions respectively. The intensity of the signal is proportional to the number of Eu ions present in the Gd_2_O_3_ nanoparticles. A very small measurable reduction in emission, and hence in mass of Eu present in a particle, is discernible after 170 hours.

**Figure 4:**
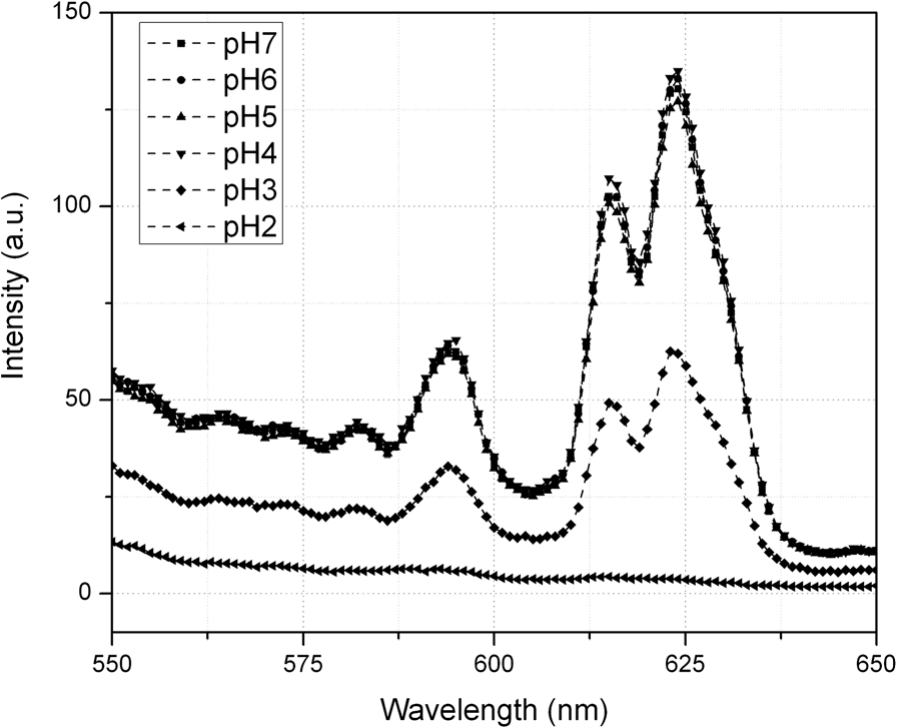
Photoluminescence spectra of Eu emission at various pH after 170 hours.

The Eu ion is luminescent following excitation when it is present in a suitable crystal host such as Gd_2_O_3_. When the ion is in solution, its luminescence is completely quenched. The photoluminescence (PL) provides an ideal measurement of the kinetics of dissolution of these particles in a range of pH conditions. The PL intensity is directly proportional to the amount of crystal-based Eu ions in suspension as illustrated in Figure 3 at pH 7 in buffer. The pH was then adjusted to study the dissolution of particles. A base line for the particle PL spectra was obtained by dissolving the nanoparticles in HNO_3_ for which we expect to see zero PL. With all the particles dissolved, indeed no measurable emission was detected. The spectra of particle PL emission were collected at several time points in each of the media of interest (lung BALF and reduced pH PBS). The results after 7 days are shown in Figure 4. The PL spectra of the nanoparticle dispersions indicate that after 7 days, very little or no dissolution of Eu ions occurred in either lung BALF or low pH buffer solution down to pH 4. At pH 3 and below, some dissolution was observed. However, such low pH conditions are not biologically relevant. Under conditions that are relevant to our studies, the particles are inert for an extended period of time.

### Intratracheal aspiration and translocation

**Figure 5:**
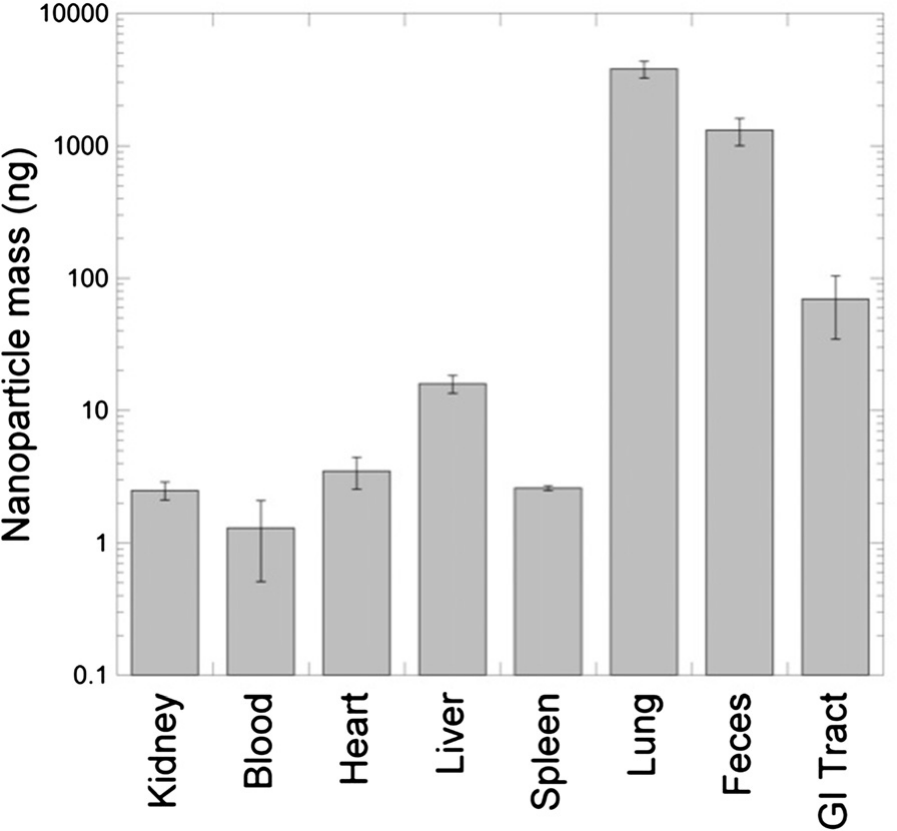
**Mass of nanoparticles found in mouse tissue samples (24 hours after instillation).** Error bars are one standard deviation (n = 4).

Measurements of the fate of instilled nanoparticles 24 hours after instillation are shown in Figure 5. Particles were detected in all the organs that were studied, although a large fraction of the instilled particles remained in the lung. A significant portion of the dose was found in the feces as shown in Figure 5, suggesting that particles were being cleared from the lung into the gastrointestinal tract. The total mass of the instilled NP was 6416 ng ± 798 ng of which 5326 ng ± 662 ng were recovered by ICP-MS data, resulting in a total delivered dose recovery of 83% (Table 1).

**Table 1:** **Mass recovery of Eu:Gd**_**2**_**O**_**3**_**nanoparticles by ICP-MS 24 hr after oropharyngeal instillation in mice**

**Tissue**	**Recovered mass (ng)**	**Percent of delivered dose (%)**
Lung	3786 ± 540	59.0
Feces	1306 ± 301	20.4
GI tract	69 ± 35	1.1
Liver	15.9 ± 2.4	0.2
Heart	3.5 ± 0.9	< 0.1
Spleen	2.6 ± 0.1	< 0.1
Kidney	2.5 ± 0.4	< 0.1
Blood	1.3 ± 0.8	< 0.1

**Figure 6:**
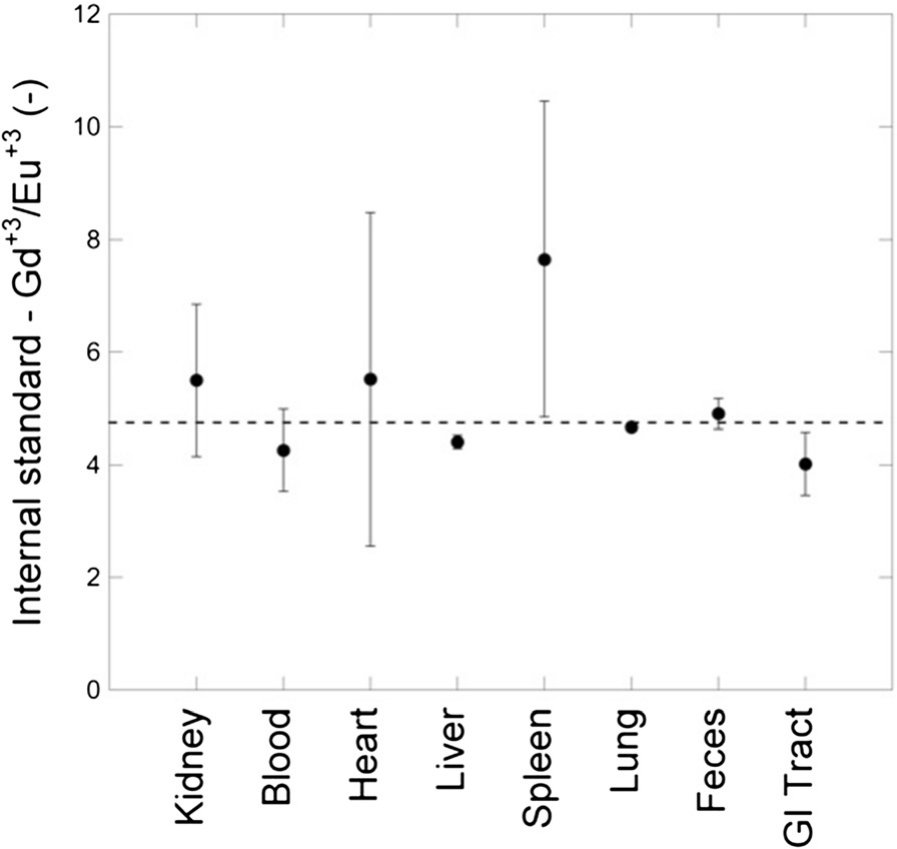
**Ratio of gadolinium to europium ions used as an internal standard as measured by ICP-MS.** Dotted line represents target value (4.74) based on initial precursor Gd/Eu ratio used in flame synthesis of nanoparticles.

We have raised the concern with regard to dissolution earlier and addressed that concern by examining the PL of the Eu ion doped into its crystal host. Our unique particle composition offers yet another check on the integrity of the nanoparticles as they interact with cells and biological fluids. If particles are intact as they translocate or clear, the ratio of Eu to Gd must remain the same. Maintenance of the original Gd:Eu ratio is a necessary, but not sufficient, condition to ensure the integrity of the particles – it is an additional confirmation that we are measuring intact nanoparticles and not dissolved ions. The ratio of gadolinium to europium ions measured by ICP-MS was analyzed for all tissue samples. The target Gd:Eu ratio was 4.74 based on the initial precursor concentrations of Gd and Eu nitrates. Figure 6 shows the measured Gd:Eu ratio. In general, the data satisfy the necessary condition for the integrity of the nanoparticles. The spleen data showed large uncertainty in the measured Gd:Eu because the concentrations of Gd^+3^ and Eu^+3^were close to the detection limit of the instrument.

A dynamic protein corona is known to form around nanoparticles suspended in physiological fluid such as lung serum [18,19]. The BCA assay for adsorbed protein indicated that the amount of BSA adsorbed per particles was 308 mg of protein per g of particles. Li et al. studied protein adsorption on flame-synthesized iron oxide and showed similar quantities of BSA (348 mg of BSA/g of particle) on the nanoparticles [20]. The approximate coverage of a monolayer of BSA on nanoparticles can be calculated [21,22] as

(2) $$S=\frac{6}{\mathrm{\rho D}}C$$

where *S* is the mass of surface-adsorbed protein per mass of particles, ρ is the particle density (7.41 g/cm^3^ for bulk gadolinium oxide), *D* = 70 nm is the spherical equivalent particle diameter, and *C* the protein surface density (0.29 μg of protein/cm^2^for BSA) – leading to *S* = 33.6 mg of protein per g of nanoparticle. A comparison with the experimental result suggests that several layers of protein, not a monolayer, coated the particles. The affinity to protein can be attributed to a negative surface charge on Eu:Gd_2_O_3_ particles (ζ = −27.7 ± 0.46 mV). Flame synthesized titanium oxide nanoparticles [23], for example, have a similar surface charge and comparable interactions with biomolecules is expected [24].

## Discussion

The use of lanthanide/rare earth doping of metal oxide nanoparticles has proven to be highly effective as a measure of dose and translocation *in vivo*. The particles are relatively simple to synthesize and the method is well suited to eventual application to inhalation studies. The very high sensitivity of ICP-MS offers a host of new possibilities for dosimetry and deposition studies. Although the basic approach is appealingly simple, we have found that in practice it is very difficult to avoid cross-contamination of samples at the very low levels that are demanded by ICP-MS detection – extraordinary care is necessary during the excision of organs and also during the digestion process, particularly during the separation of control tissues from exposed tissues. Our overall mass balance, based on the true mass input of lanthanides versus the mass recovered, is 83% ± 10% (Table 1). It is very likely that some of the lanthanide that is unaccounted for is present in the fur or carcass that was not digested. The syringe dead volume, used to instill lanthanide NP, was measured gravimetrically and resulted in a decrease of the delivered dose by 2.7% ± 0.7%; this was accounted for in the mass balance. Feces were collected from the bedding and although extreme care was taken to retrieve almost the entire sample, incomplete recovery could be another source of error. Urine samples studied using metabolism cages (data not shown here) did not indicate the presence of lanthanide ions by ICP-MS. In this study, no assumptions were made regarding the rates of fast clearance; 21.4% of the instilled dose was recovered from the feces and GI tract. A translocation study of 20 and 80 nm radioactive iridium particles instilled in rats and mice showed 95% total mass recovery with typical uncertainties related to radiological measurements [9]. Semmler-Behnke et al. reported close to 100% mass recovery for 1.4 and 18 nm gold particles intratracheally instilled in rats [25]; the mass removed from the lung by fast clearance was estimated (up to 25%) and subtracted from the delivered dose. The total mass balance determined in this work is comparable to these previous studies, within experimental error. Clearly, particle size also determines ability to translocate. Kreyling et al. [9] found that iridium particles of 15 nm were much more capable of translocating from the lung tissue than 80 nm particles.

The number-weighted mean diameter of our particles in suspension in buffer, as delivered to the animals, was 84 nm with a relatively narrow size distribution as measured by dynamic light scattering. We are able to vary this size in our method by controlling independently two parameters: (1) precursor concentrations in the solutions, the easiest parameter to change; (2) the size of droplets formed in our spray. Inhalation experiments that will follow on from this methodology study will examine this important issue.

As shown in Table 1, 24 hours after nanoparticle instillation, 59% of the initial dose was measured in the lung, 20% was excreted in the feces, 0.2% was detected in the liver and less than 0.1% of the delivered dose was detected in the remaining organs. These results are qualitatively similar to those found by Kreyling et al. [9] for 15 and 80 nm iridium particles intratracheally instilled in adult rats; after 7 days, 59% of the delivered dose was found in the lungs, 35% was fecally excreted and less than 0.2% found in extrapulmonary organs. In contrast, studies of 40 and 100 nm gold nanoparticle that were instilled intratracheally did not yield measurable signal in the liver or other organs [26]. This could be due to different protein coating on gold compared to Eu:Gd_2_O_3_ nanoparticles or comparable flame-generated metal oxide particles.

It is possible that metals are not transported in animals as nanoparticles but rather as dissolved ions, which could have a quite different propensity for translocation and clearance. Our particles offer yet another advantage: a straightforward means to estimate the rates of dissolution within physiologically relevant media. Our ability to track dissolved versus bound Eu in a particle via photoluminescence offers a way to determine the importance of nanoparticle dissolution. We have found very little evidence of dissolution after 7 days in either of the simulated fluids that particles may encounter: lung BALF and low pH buffer, the latter designed to simulate a lysosomal condition. Further evidence for this assertion comes from examination of the Gd:Eu ratio in recovered tissues – the ratio of the metal ions in the measured samples remains the same. It is unlikely that these ions would diffuse at precisely the same rate, therefore, the ratio measurements indicate that it was likely that the elements translocated together within the particle and that this is the primary contributor to the observed clearance and translocation processes that we have measured.

This experimental method promises to provide new insights into clearance and deposition, with a quantifiable dose. Dose at the target site is proportional to biologic effect – this is a basic tenet of toxicology. Yet when it comes to inhalation dosimetry, air pollution standards and most animal exposures characterize the concentration of particles in the air on the basis of mass per volume as the “dose”. This does not account for the actual mass deposited into the initial target tissue – the respiratory tract – which can be affected by disease, age, exercise etc. Local toxic effects are of special interest for ultrafine particles because they have a larger total surface area for an equivalent mass of larger particles and a long retention time in the lung. While there has been recent notable progress in using computational models to predict total respiratory tract deposition, confirmation of the models with experimental data has lagged [27]. If we could inexpensively, reliably, and with great sensitivity, track inhaled particles in the body, it would revolutionize the field of inhalation toxicology and allow the interpretation of biological response in relation to delivered, and retained, dose.

While radionuclides have shown promise for this purpose, with sensitivity in the range of 1 ppm [9,28,29], issues of containment and cleanup have limited their usefulness. Stable isotopes exhibit some promise [30]. Fluorescent beads [31] and quantum dots are useful for short term studies, particularly intracellular tracking studies, but issues of photo-bleaching and toxicity [32], respectively, can limit their usefulness. The particles described in the current study are a breakthrough in this respect because they are synthesized as part of a flame pyrolysis process and so lend themselves readily to inhalation exposure studies. Furthermore, they can be fine-tuned into specific size ranges by altering spray droplets sizes and the concentrations of precursors in solution.

We chose to use oropharyngeal aspiration for these initial studies because dose can be readily controlled and the particles delivered as a relatively large bolus, which is desirable for studies of translocation and testing of the range of detection. We acknowledge that this method does not approximate a true inhalation exposure and is not appropriate for studies of short-term clearance, which would be more appropriately addressed using an inhalation model [6,33]. Furthermore, some aggregates of particles were detected and this can affect deposition and translocation characteristics, but these aggregates were a small percentage of the total number of particles instilled. This is a limitation of the instillation approach and will be addressed in future studies of aerosols.

Particle clearance can be thought of in two phases. The first phase occurs primarily in the 24 hours after dosing and is dominated by conducting-airway mucociliary clearance of the particles to the larynx and then to the GI tract and feces [28]. The second phase, which is thought to involve macrophage-mediated clearance from the peripheral lung, occurs after 24 hours and can last for a long time (hundreds or thousands of days) depending on the particle type [33]. The major issue with particle tracking following respiratory tract deposition is that only a very small percentage of the particles escape the lung and circulate to other organs (this varies based on particle size and has been estimated as < 0.1% for particles in our size range [28]). This means that for a method to be useful for particle tracking, it needs to be exquisitely sensitive to a very small number of particles.

Our approach using Eu:Gd_2_O_3_ particles can detect particle loads in the ppb range. However, simply estimating particle mass in a whole organ will likely not be sufficient; the logical next step for biological investigations will be to define the extracellular and intracellular location of the particles, preferably with the same particles from the same exposure that characterized the local dose. This is the goal of future studies and will be facilitated by the native phosphorescence of these particles that will allow them to be visualized microscopically.

## Conclusion

The translocation of aspirated particles in mice was studied using flame-synthesized europium-doped gadolinium oxide nanoparticles. The particles were characterized for size, surface charge and morphology. The flame-synthesized particles were a good surrogate for typical metal oxide ultrafine particles that can be produced by a variety of synthesis routes. Particle dissolution in typical biological media was evaluated using photo-luminescent emission and was found to be insignificant over the 24 hour period of this study. The majority of the particles remained in the lung after 24 hours. Particles were also detected in the gastro-intestinal tract and feces suggesting a fast clearance of some of the particles out of the lung. Small but detectable signals were quantified in all the organs that we studied with the highest concentration found in the liver. Negligible amounts were found in the blood, suggesting clearance from the blood by other organs. The combination of metal oxide nanoparticles doped with a rare earth element, along with ICP-MS elemental analysis, provides detection limits in the low ppb range. The fact that the nanoparticles can be generated in an aerosol process opens up the possibility that this approach can be used successfully in an inhalation exposure study. The particles and approaches described here can be used in future studies, in combination with site specific methods we have developed to study lung biology [34] and inhalation exposures, to determine site specific deposition within the respiratory tract, both quantitatively and qualitatively. We can then, for the first time, link dose with biological effect.

## Methods

### Particle synthesis

**Figure 7:**
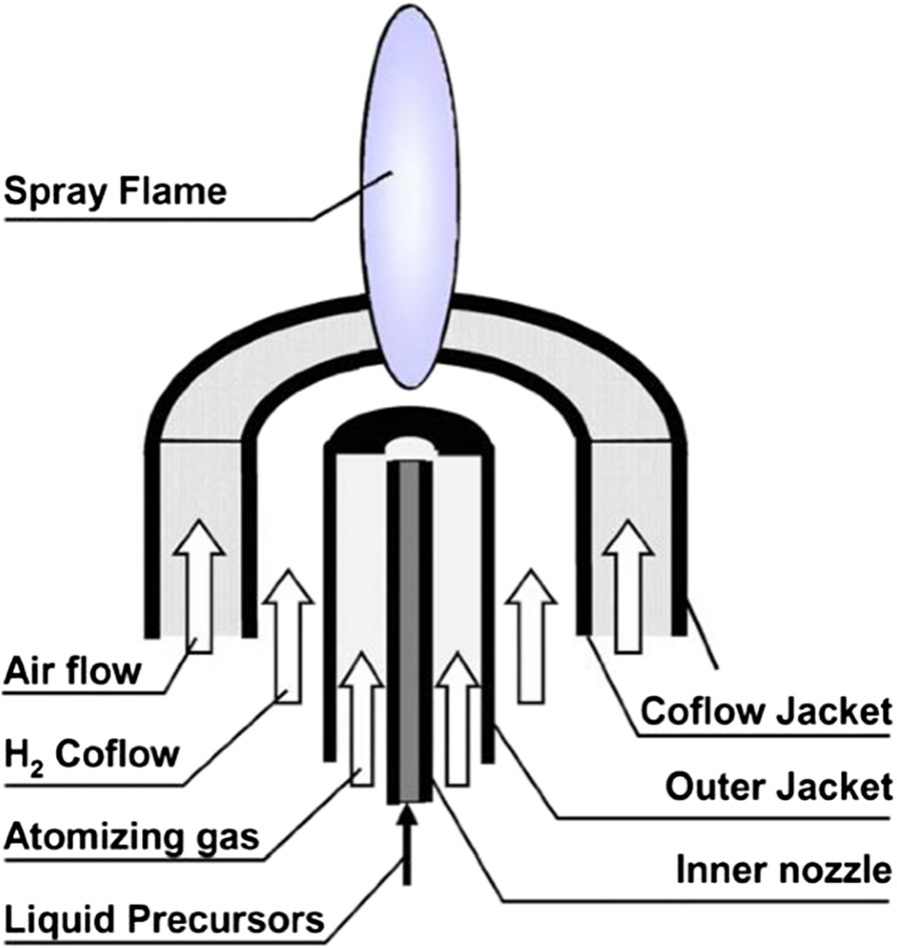
**Flame spray pyrolysis burner schematic used for synthesizing Eu:Gd**_2_**O**_3 _**nanoparticles.**

Europium doped gadolinium oxide nanoparticles were synthesized using a flame spray pyrolysis technique and a forced jet atomizer similar to that described by Dosev et al. [35]. The burner schematic is shown in Figure 7. This technique is well-suited for generating environmentally relevant metal oxide nanoparticles in high concentrations with reasonable control on particle size [36]. Gadolinium and europium nitrate salts (35.5 mM and 9.3 mM respectively) were dissolved in ethanol and used as the liquid precursor. The precursor was sprayed into a hydrogen-air flame at 40 ml/hr using a syringe pump. The precursor droplets were pyrolyzed to form nanoparticles in the high temperature environment. The Gd:Eu ratio measured by ICP-MS was 4.7 ± 0.1 and in agreement with the initial precursor ratio (4.74) based on nitrate concentrations in the precursor. Particles were collected on a cold finger by thermophoresis; the collected powder was washed in Milli-Qultrapure (MQ, 18.3 MΩ-cm) water to remove any unreacted precursor from the nanoparticles. The nanoparticles were dried and stock solutions of 10 mg/mL were prepared in MQ water for the instillation experiment.

### Particle characterization

The nanoparticle morphologies were investigated using a Phillips CM-12 transmission electron microscope (TEM) operated at 120 kV. Nanoparticles were suspended in MQ water and deposited on a 400 mesh copper TEM grid with a carbon/Formvar® film (Ted Pella Inc. Redding CA. Prod # 01754-F); excess liquid was wicked away.

The particle crystalline phase was identified using a Scintag powder x-ray diffractometer (XRD) with Cu Kα radiation operated at 45 kV and 40 mA.The powder was scanned for 2θ = 25° calculated using Scherrer’s formula,

(3) $$D_{\mathrm{XRD}}=\frac{\mathrm{K\lambda}}{\beta \cos\theta }$$

where the shape factor *K* is 0.9 [37], λ is the incident x-ray wavelength (=1.54 Å), β is the peak full width-half maximum (FWHM) at Bragg angle θ = 32.5°.

The surface area of nanoparticles was measured with the Brunauer-Emmett-Teller (BET) method using an Autosorb-1 instrument (Quantachrome Instruments, Boynton Beach, FL). A sample of 380 mg was measured with nitrogen as the adsorbate. Hydrodynamic particle size measurements were performed using a BIC 90Plus dynamic light scattering instrument

(Brookhaven Instruments, Holtsville, NY). The number-weighted (NW) particle size distributions were calculated by the 90Plus software (Brookhaven Instruments). The particle concentration at the start of the DLS experiment was 20 mg L^-1^ in MQ water. The solution was bath-sonicated for one minute to disperse the particles. At least five runs were measured and the results were averaged.

Zeta potential was measured by light scattering using a BIC ZetaPlus instrument (Brookhaven Instruments Corporation, NY). Particles were suspended in 1 mM KCl with particle concentration of 100 mg L^-1^ and the suspension was bath-sonicated for five minutes before each sample measurement. At least five measurements were made for each sample and the data were averaged.

### Surface adsorbed protein quantification

Particles were suspended in either PBS (1x) or BSA (100 mg/mL of dry protein in PBS (1x)) with particle concentrations at 200 mg L^-1^. Particles were washed in PBS (1x) three times. The solutions were bath-sonicated for five minutes, centrifuged at 9500 g ; the supernatant was replaced with PBS between washes to remove any unbound protein. Surface adsorbed proteins were quantified using the bicinchoninic acid (BCA) assay with bovine serum albumin (BSA) as a calibration standard. The absorption measurements (excitation wavelength, λ = 560 nm) were performed on a SpectraMax M2 cuvette/microplate reader (Molecular Devices Inc, Sunnyvale CA) using the SOFTmax PRO software. Samples were bath-sonicated before measurements. The measurements were made with six replicates. A 96-well plate was used with 200 μL in each well. After the reagent was added, the well was incubated at 37°C for 30 minutes. The calibration range extended between 20 to 500 μg/mL of BSA with R^2^ linearity of 0.998. The adsorbed protein concentration was determined by subtracting the signal of uncoated bare particles from protein-coated particles.

### Particle dissolution

We investigated the possibility that Eu and Gd ions are translocated, not as nanoparticles, but as dissolved ions. It is important to estimate the rate of dissolution of these particles under conditions that approxim ate the fluid in the lung and also the intracellular conditions of an endosome. To obtain sufficient lung lavage fluid for in vitro experiments of particle dissolution, lung bronchoalveolar lavage fluid (BALF) was obtained from adult Sprauge-Dawley rats (N = 6), instead of the mice that were used for the translocation studies. Briefly, rats were euthanized with an overdose of pentobarbital given i.p., the trachea cannulated and a single volume of 35 ml kg^-1^ of buffered saline solution was lavaged into the lung three times. The physiological conditions that are typical of a cellular lysosome were modeled with PBS adjusted to a pH of 5.

The Eu^3+^ ions in Gd_2_O_3_ emit light only when contained within the host matrix. Any dissolution or leaching of Eu^3+^ ions from the host Gd_2_O_3_ nanoparticle will result in a drop in the photo-luminescence (PL). The PL spectra of particle suspensions were obtained using a Varian Cary Eclipse Fluorescence Spectrophotometer equipped with a Xenon lamp as an excitation source. The dry nanoparticles samples were dispersed in solutions of lung serum and PBS at pH5 and sonicated for 15 min to obtain a transparent dispersion of nanoparticles with a concentration of 200 μgml^-1^. In each case, 4 ml of the nanoparticle dispersion were added to a quartz cuvette and excited at 250 nm to obtain the emission spectra of the nanoparticles over time. To investigate the dissolution of the nanoparticles at different pH, PBS solutions were adjusted to different pH (i.e. 2 to 7) and emission the spectrum were obtained using the same method.

### Oropharyngeal aspiration

#### Animals

All animal experiments were performed under protocols approved by the University of California Davis Institutional Animal Care and Use Committee in accordance with NIH guidelines. For studies of particle translocation in vivo, adult (8 weeks) male NIH Swiss mice with a weight of 25 to 30 grams were purchased from Harlan Laboratories (Livermore, CA). Mice were delivered one week prior to exposure, and housed in AALAC approved facility at the Center for Health and the Environment, University of California Davisand provided with Laboratory Rodent Diet (Purina Mills, St. Louis, MO) and water *ad libitum*.

#### Oropharyngeal aspiration of particles

Oropharyngeal aspiration in mice is an attractive alternative to direct tracheal instillation because it results in less variability among animals and gives a more uniform pulmonary distribution of the administered particles [38]. Mice were anesthetized using a Quantiflex anesthesia machine (Midmark Corp., Versailles, OH) equipped with an isoflurane vaporizer. Mice were placed in a Plexiglass® box connected to anesthesia machine. A mixture of 2.5% isoflurane and oxygen was delivered at a rate of 1 L/min to effect, approximately five minutes. Once anesthetized, a known concentration of particles (40 μL dose of 0.160 ± 0.02 g L^-1^ Eu/Gd PM suspended in MQ water) was pipetted into the oropharynx and aspirated into the lungs [39]. Mice were monitored until recovery. The dead volume of the instillation system (Product # RSPSMI, Kent Scientific, CT) was determined gravimetrically. Following recovery from anesthesia, each mouse was placed in an individual cage. N = 4 per group

#### Necropsies and organ harvest

Tubes were weighed before and after sample was collected. Mice were euthanized an overdose of pentobarbital (150 mg/kg) given i.p and exsanguinated. Blood was collected and the abdominal cavity was opened and the spleen, kidneys and liver were removed. Then the thoracic cavity was opened and the heart and lungs were removed. Finally, the stomach and intestines were removed as a unit. Feces from the cages were collected and the remaining carcass was weighed and frozen at −80°C. Minced, tissues, blood and feces were placed in individual 15 mL polystyrene conical tubes (BD Biosciences, Franklin Lakes, NJ) for processing. Instruments were cleaned in DI water and rinsed in 70% ethanol between uses to prevent cross contamination.

#### Tissue acid digestion and ICP-MS analysis

Tissue samples were digested in trace metal grade nitric acid and hydrogen peroxide for elemental analysis. Acid digestion occurred at 70°C for 24 hours followed by hydrogen peroxide (70°C) digestion overnight. Tissue samples were diluted with MQ water to an acid concentration of 6%.

The concentrations of elemental gadolinium and europium were quantified using inductively coupled plasma mass spectrometry (ICP-MS). The ICP-MS instrument was calibrated using a NIST traceable standard for Eu and Gd. The standards came in stock solution at 1000 ppb and 100 ppb; serial dilutions of 500, 200 and 100 ppb were used at the high end of the concentration range and 100, 10, 1 and 0.1 ppb for the low concentration standard to generate the calibration curve. The instrument level of detection (LOD) was 1.9 ppt (parts per trillion) for Eu and 2.7 ppt for Gd and the BEC (background equivalent concentrations) is 1.6 ppt for Eu and 3.9 ppt for Gd. The LOD and BEC are determined by instrument rinses with at least n = 5 throughout the experimental run on a particular day. Blank tissues were used as a control; the ICP-MS analysis of Eu indicated a maximum concentration in these tissues of 0.1 ppb, setting a lower limit to our sensitivity in the exposure experiments that translates to a limit of quantification of about 4 ng in the lung samples and about 1 ng in the kidneys.

Quality control of the ICP-MS analysis and the integrity of nanoparticles was checked by comparing the measured Gd:Eu ratio from tissue samples to that of the original nanoparticles, ensuring that the elemental signal was from nanoparticles and not from the background, and that analytical artifacts had not been introduced. Indeed, europium-doped gadolinium oxide is suitable for this experiment as both elements have a low natural abundance abundance. The most common rare earth element is Cerium with a natural abundance in the Earth’s crust of about 43 ppm [40]; the least common is Thulium at 0.3 ppm. Europium and Gadolinium are present in the Earth’s crust at around the 1 ppm level. Good hygiene in the laboratory ensures that the background levels in our experiments are well below the natural level with our controls exhibiting concentrations at about 0.1 ppb.

## Competing interests

The author(s) declare that they have no competing interests.

## Authors’ contributions

AA designed the study, synthesized the nanoparticles, and undertook the ICP-MS analysis, as well as drafting the manuscript. DA performed the animal experiments and participated in writing the manuscript. GD carried out the dissolution studies and participated in writing the manuscript. LV supervised the animal experiments and participated in writing and editing the manuscript. IK conceived of and designed the study, supervised the synthesis of nanoparticles, and participated in writing the paper. All authors read and approved the final manuscript.
